# Magnitude of adolescent sexual activity and its associated factors among selected secondary school student in Addis Ketema Sub-city, Addis Ababa, Ethiopia

**DOI:** 10.1093/sexmed/qfag044

**Published:** 2026-06-27

**Authors:** Elias H Babeta, Hussen M Asfaw, Getahun T Dibaba

**Affiliations:** Department of Public Health, Santé Medical College, Addis Ababa, Ethiopia; Department of Reproductive Medicine, Addis Ababa University, College of Health Sciences, Addis Ababa, Ethiopia; Departments of Medical Physiology, Addis Ababa University, School of Medicine, College of Health Sciences, Addis Ababa, Ethiopia

**Keywords:** adolescent sexual activity, adolescent, magnitude, sexual practice, Addis Ababa

## Abstract

**Introduction:**

Early sexual debut poses significant risks to adolescent health. This study investigated the prevalence and factors associated with adolescent sexual activity among secondary school students in Addis Ketema, Addis Ababa, Ethiopia.

**Methods:**

A quantitative cross-sectional study was conducted using self-administered questionnaires from November to December 2021. Data were analyzed using frequency distributions and logistic regression. The primary outcomes were the prevalence of adolescent sexual activity and the adjusted odds ratios (AOR) for its significant associated factors.

**Results:**

The prevalence of adolescent sexual activity was 17.3%, with a majority being male (77%). The most common reason for initiation was self-desire (36.1%). Significant predictors were having a boyfriend/girlfriend (AOR = 4.58), alcohol consumption (AOR = 5.42), school type (AOR = 3.15), and watching pornography (AOR = 21.25).

**Discussion:**

A considerable prevalence of adolescent sexual activity exists in this setting, driven by identifiable relational, behavioral, and environmental factors. Key strengths include direct measurement of a sensitive public health issue. Limitations comprise potential social desirability bias in self-reported data, limited qualitative depth, and possible underreporting due to parental consent requirements for minors. Targeted interventions should address modifiable risk factors, particularly pornography consumption and substance use, to reduce early sexual activity.

## Introduction

Adolescent sexual activity remains a critical public health concern, particularly in low- and middle-income countries, where over 1.2 billion young people aged 15-24 reside—more than 200 million in sub-Saharan Africa.^1^ In Ethiopia, this age-group constitutes over 20% of the national population. Adolescence is a transitional period marked by physical, cognitive, and social maturation, often accompanied by increased risk-taking, sexual curiosity, and reduced parental guidance, which can lead to unprotected sexual activity with serious health consequences including unintended pregnancy, sexually transmitted infections, and HIV.^1–4^ Globally, the prevalence of adolescent sexual activity varies widely due to sociocultural factors, yet sub-Saharan Africa bears a disproportionate burden.^1^^,^^2^

In Ethiopia, studies among secondary school students report varying prevalence rates: 28.2% in Jimma, 22.5% in Debre Tabor, 31.3% in Debre Markos, 17.6% in Adigrat, and 19.8% in Addis Ababa.^5–9^ Research in Goba town found a rate of 31.2%, with older students and those lacking reproductive health knowledge at higher risk.^10^ Similarly, a study in Nekemte reported that 48.6% of youths had engaged in sexual activity, most before age 18.^11^ These behaviors are often unprotected, exposing adolescents—particularly girls—to heightened biological, economic, and social vulnerabilities.^12^

Multiple factors influence adolescent sexual activity beyond individual behavior. Key predictors include sociodemographic characteristics (age, sex, family structure), substance use (alcohol, khat), peer pressure, and exposure to sexually explicit media.^3^^,^^8^^,^^9^^,^^13^^,^^14^ Adolescents not living with both biological parents are more than twice as likely to be sexually active,^4^ and economic pressures—both poverty and desire for material goods—have been identified as drivers, especially among girls.^3^

Addis Ketema sub-city, 1 of the oldest and most densely populated areas of Addis Ababa, is a major commercial and transportation hub attracting migrant and transient populations. This environment creates a unique social fabric where traditional family structures coexist with more permissive norms, potentially intensifying peer influence, economic pressures, and reduced parental oversight compared to other parts of the city.

Given the rapidly growing adolescent population in Ethiopia and the long-term health implications of behaviors established during this stage, location-specific data are urgently needed to inform targeted interventions. While existing evidence indicates that adolescent sexual activity is a significant concern in urban Ethiopian settings, the precise magnitude and contributing factors within Addis Ketema sub-city remain insufficiently characterized. To address this gap, this quantitative study was designed to answer the following refutable question: Is there a significant association between individual, relational, behavioral, and environmental factors and self-reported sexual activity among secondary school students in Addis Ketema sub-city, Addis Ababa, Ethiopia?

### Conceptual framework (Figure 1)

**Figure 1:**
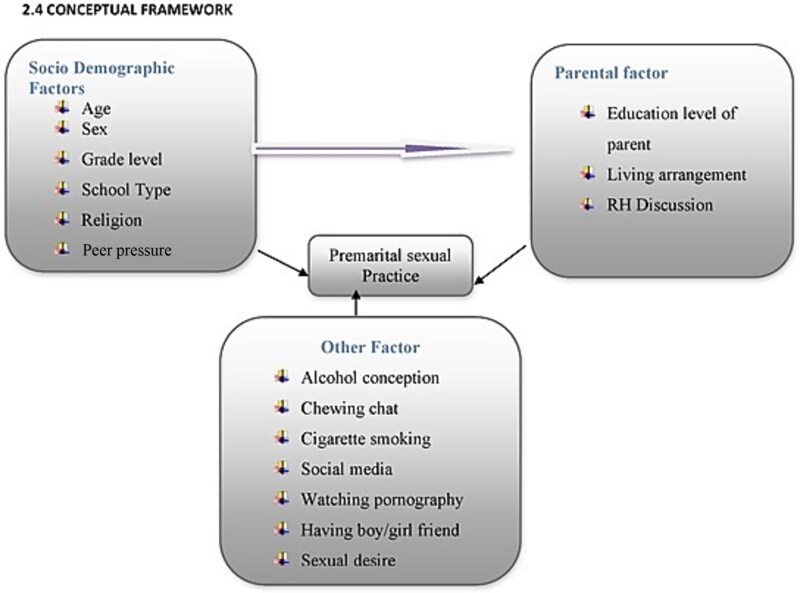
Conceptual framework of magnitude of adolescent sexual activity and associated factor (6, 15).

#### Objectives

##### General objective

To assess the magnitude of adolescent sexual activity and associated factors among selected secondary school students in Addis Ketema sub-city.

##### Specific objectives

- To determine the magnitude of adolescent sexual activity among selected secondary school students in Addis Ketema sub-city.
- To assess associated factors of adolescent sexual activity among selected secondary school students in Addis Ketema sub-city.

## Methods

### Study area

The study was conducted in Addis Ketema sub-city, 1 of the 11 sub-cities of Addis Ababa. The largest open market in the city is found in this sub-city. It is located in the northern part of Addis Ababa, bordered by Gullele in the north, Arada in the east, Kolfe Keranio in the west, and Lideta in the south. There are 5 government schools and 3 private secondary schools in the sub-city. In 2021, the total number of adolescent students was 6586.^2^^,^^3^

### Study design

A school-based cross-sectional study was used to assess the magnitude of adolescent sexual activity and associated factors among selected secondary schools in Addis Ketema sub-city.

### Study period

The study was conducted from November to December 2021.

### Source population

All Addis Ketema sub-city government and private secondary school students during the study period.

### Study population

All regular daytime students attending selected secondary schools of Addis Ketema sub-city during the data collection period who fulfilled the inclusion criteria.

### Inclusion criteria

All regular students who were in grades 11 and 12 during the 2021/2022 academic year and aged 15-24 years from government and private secondary schools were included.

### Exclusion criteria

Students who were absent during data collection or who were critically ill during data collection were excluded.

### Sample size

The sample size was determined using the formula for a single population proportion, taking the proportion from a study conducted in Debre Markos town, northwest Ethiopia, where the magnitude of adolescent sexual activity among preparatory school students was 31.3%,^1^ with a 95% CI (*Z* = 1.96) and a margin of error of 5% (*d* = 0.05). Computing with the above formula and adding 10% for nonresponse gave a total sample size of 330; after adding the 10% nonresponse rate, the final sample size was 360 (Table 1).

**Table 1:** Sample size for factor associated with expose sexual activity among selected secondary school student in Addis Ketema sub-city, Addis Abeba, 2021 by using Epi Info.

**Variables**	**Exposed primordial sexual practice**	**CI**	**Power**	**OR**	**Total sample size**	**Reference**
Peer pressure	9.3	95	80	7.65	60	Study contract on Debre Markos, Hossana Town
Watching pornography	51	95	80	4.93	74	
Drinking alcohol	34	95	80	6.62	48	

The formula used:

n = (*Zα*/2)2 × *P*(1 − *P*)*d*2n = *d*2(*Z*α/2​)2 × *P*(1 − *P*)​

where:

*P* = .313 (from previous study in Debre Markos^1^)

*d* = 0.05

*Zα*/2 = 1.96

n = (1.96)2 × 0.313 × 0.687(0.05)2 = 330n = (0.05)2(1.96)2 × 0.313 × 0.687​ = 330

330 + 10% nonresponse = 360

Because the sample sizes calculated for associated factors were smaller than that for the magnitude, the sample size was based on the single population proportion for magnitude (330) plus 10% nonresponse, yielding 360.

### Sampling procedure

Addis Ketema sub-city was purposively selected for being the smallest and most densely populated. From its secondary schools, 40% were chosen via stratified sampling to represent both government and private institutions, resulting in 2 government schools and 1 private school being selected randomly.

The total sample size of 360 was proportionally allocated to each of the 3 selected schools based on their student population size using the formula:

nj = nN × Njnj​ = Nn​ × Nj​

where:

n_j = sample size for the selected school

N_j = total population of that school

n = total sample size (360)

N = total number of students in all selected secondary schools (2828)

- **Dilachen Secondary School:** 360 × 770/2828 = 98
  (Grade 11: 98 × 705/770 = 89; Grade 12: 98 × 65/770 = 9)
- **Yekatit 23 Secondary School:** 360 × 1725/2828 = 220
  (Grade 11: 220 × 735/1725 = 94; Grade 12: 220 × 990/1725 = 126)
- **Radical Secondary School:** 360 × 333/2828 = 42
  (Grade 11: 42 × 133/333 = 16; Grade 12: 42 × 200/333 = 26)

**Figure 2:**
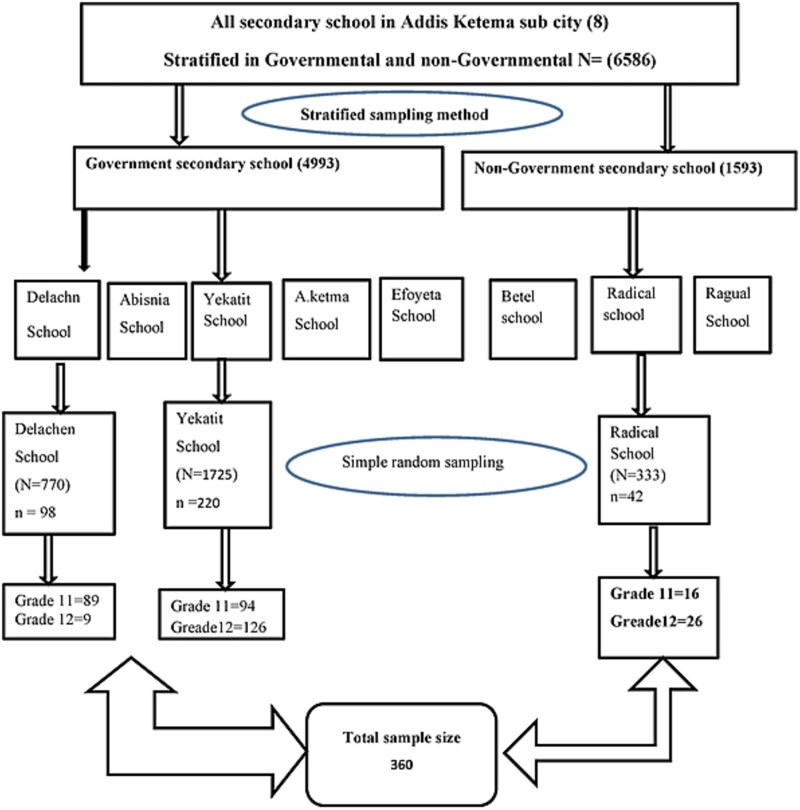
Sampling procedures of magnitude of adolescent sexual activity and associated factor among secondary school student in Addis Ketema sub-city, 2021.

Within each grade, students were selected using computer-generated random sampling (See Figure 2 for the sampling flow diagram).

### Operational definitions

- **Adolescents:** Individuals aged 10-19 years.^4^
- **Secondary school:** Grades 11 and 12 students attending regular classes.
- **Adolescent sexual activity:** Penetrative vaginal sexual intercourse (the term “premarital” is not used, as the study focuses on sexual activity regardless of marital status).
- **Substance use:** Use of at least 1 of the following: alcohol, khat, or cigarettes, assumed to affect thinking and increase risk of sexual activity.^11^
- **Peer pressure:** Self-reported response “yes” to the question, “Did your friend initiate you to have sex?”^1^
- **Pornography:** Films intended for sexual excitement.^11^

### Study variables

- **Dependent variable:** Sexual activity.
- **Independent variables:** (1) Sociodemographic (age, sex, grade level, religion, marital status, parental education, pocket money); (2) recreational/behavioral (alcohol drinking, khat chewing, smoking, watching pornography, peer pressure).

### Data collection procedures

Data were collected using a self-administered questionnaire adapted from existing literature and modified to align with the research objectives.^6^^,^^15^ The instrument was initially prepared in English, translated into the local language (Amharic), and then back-translated into English by a language expert to ensure consistency.

The research team consisted of 3 clinical nurses (diploma holders) as data collectors and 1 BSc health officer as a supervisor. The principal investigator coordinated all study activities. A 1-day training session was provided for the data collectors and supervisor. One week before the actual data collection, the questionnaire was pretested for clarity and consistency at a secondary school in Addis Ketema that was not part of the final sample. Throughout data collection, close supervision was maintained. The supervisor checked all completed forms for completeness, accuracy, and consistency, providing immediate feedback to the data collectors.

### Data quality assurance

Data collectors and supervisors received a 1-day training session on data collection and measurement techniques. A pretest of the questionnaires was conducted before the actual data collection to assess response accuracy, estimate the required time, and make necessary modifications; data from the pretest were not included in the main study.

Collected information was reviewed daily, and any identified errors were returned to the data collectors for immediate correction. During data processing, Epi Data and SPSS software were used to identify out-of-range values and inconsistencies.

### Data analysis

All completed questionnaires were verified for completeness. Data were entered into Epi Data version 3.1 (Epi Data Association) and subsequently transferred to SPSS version 23 (IBM Corp.) for analysis. The data then underwent cleaning, coding, and preparation for final analysis.

Descriptive statistics were employed, with means and standard deviations for continuous variables. Frequency distributions, charts, and tables were used to present categorical variables. The association between independent variables and the outcome was first assessed using binary logistic regression. All variables with a *P*-value <.25 in bivariate analysis were included in a multiple logistic regression model to control for potential confounders. Factors were considered statistically significant predictors of adolescent sexual activity if they had an adjusted odds ratio (AOR) with a 95% CI not including 1 and a *P*-value <.05.

### Ethical consideration

Ethical approval was obtained from the Research Ethics Committee of the institution’s Department of Public Health (Reference No. REC/SMC/021/2021). An official permission letter was submitted to the Addis Ketema sub-city Education Office and the respective school directors. School vice-directors and teachers were also informed, and their cooperation was requested.

Before data collection, the purpose of the study was explained to all participants. Written informed consent was obtained from participants aged 18 years and above. For minors under 18, parental/guardian written consent was obtained, followed by written assent from the adolescents themselves. All participants were clearly informed about the study’s aims, potential benefits and risks, the importance of their participation, the confidentiality of their data, the voluntary nature of their involvement, and that refusal to participate would not affect their academic standing. The study was conducted in accordance with the Declaration of Helsinki.

### Dissemination plan

The results of the study will be submitted to the institution’s Department of Public Health, the Addis Ketema sub-city Health Office, the schools, and other concerned bodies.

## Results

### Sociodemographic characteristics of the study participants

Of the 360 selected secondary school students, 353 completed the questionnaire (response rate 98%).

The mean age was 17.98 years (SD = 0.95; range 16-21). The majority (88.4%) were from government schools, and 55% were in grade 11. Females comprised 51.6% of the sample. Most participants identified as Orthodox Christian (60.3%), and 47.9% reported receiving pocket money. Regarding living arrangements, 91.8% lived with their family. In terms of parental education, 5.9% of fathers and 11.3% of mothers were reported to be illiterate (Table 2).

**Table 2:** Sociodemographiccharacteristics of secondary school student in Addis Ketema sub-city, Addis Ababa, 2021 (*N* = 6586).

**Variable**	**Characteristic**	**Frequency**	**Percent (%)**
Sex	Male	171	48.4
	Female	182	51.6
Age	15-17	100	28.3
	18-21	253	71.7
Grade	11	194	55.0
	12	159	45.0
Religion	Orthodox	213	60.3
	Muslim	102	28.9
	Protestant	31	8.8
	Catholic	7	2
School	Private	41	11.6
	Government	312	88.4
Pocket money	Yes	169	47.9
	No	184	52.1
Living arrangement	Family	326	92.4
	Relative	21	5.9
	Alone	6	1.7
Education status of father	Illiterate	21	5.9
	Literate	79	22.4
	Elementary	84	23.8
	Secondary and above	169	47.9
Education status of mother	Illiterate	40	11.3
	Literate	72	20.4
	Elementary	105	29.7
	Secondary and above	136	38.5
Occupation status of father	Government	103	29.1
	Private	138	39.1
	Daily labor	28	7.9
	Merchant	84	23.8
Occupation status of mother	Government	54	15.3
	Private	83	23.5
	Daily labor	16	4.5
	Merchant	55	15.6
	Housewife	145	41.1

### Objective 1: Magnitude and characteristics of adolescent sexual activity

#### Prevalence of adolescent sexual activity

A total of 61 adolescents reported ever having engaged in sexual intercourse, yielding an overall prevalence of **17.3%** (95% CI, 13.5-21.6). Among these, 77% were male, and 50.8% had been sexually active within the past 12 months. The median age at first sexual intercourse was 18 years.

#### Condom use and reasons for sexual initiation

**Figure 3:**
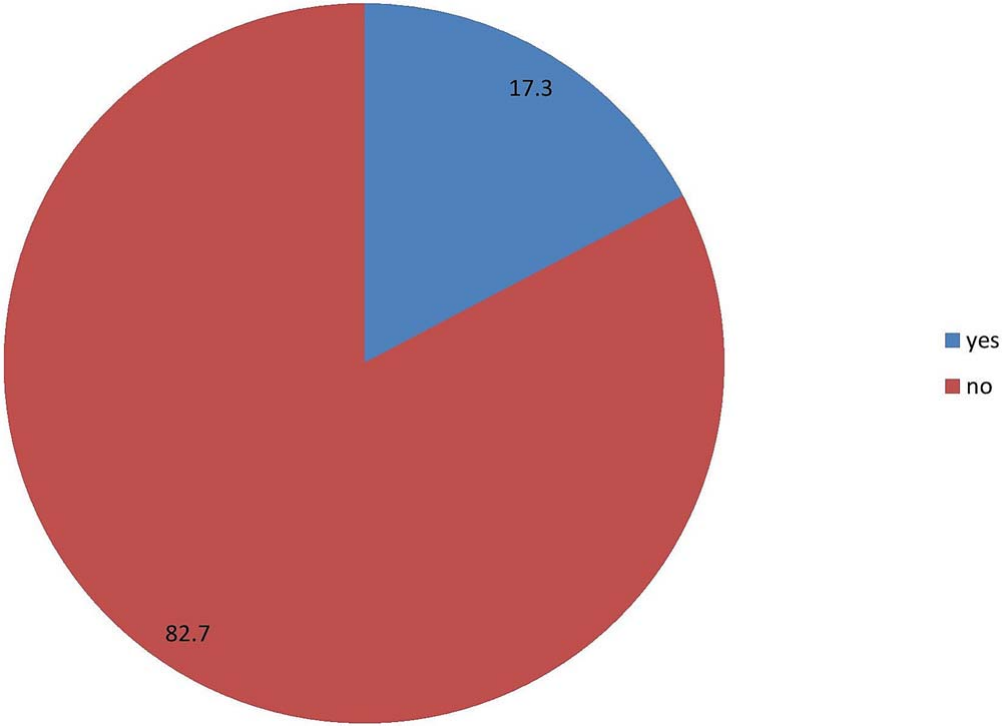
Magnitude of adolescent sexual activity of secondary school student in Addis Ketema sub-city, 2021.

Among sexually active students, 72.1% reported using a condom during their first sexual encounter. The primary reason cited for sexual initiation was self-desire (36.1%), followed by having a boyfriend/girlfriend (29.5%) and peer pressure (24.6%). Regarding sexual partners, 31.1% reported having had sex with a fellow student (Table 3 and Figure 3).

**Table 3:** Experience of sexual activity of secondary school student in Addis Ketema sub-city, Addis Ababa, 2021.

**Variable**	**Characteristics**		**Percent (%)**
Having boy or girlfriend	Yes	182	51.6
	No	171	48.4
Sexual practice	Yes	61	17.3
	No	292	82.7
Reason for sex	Having boy/girlfriend	18	29.5
	Self-desire	22	36.1
	Peer pressure	15	24.6
	To get economic benefit	6	9.8
Sexual partner	Student	29	47.4
	Boy or girlfriend of respondents	27	44.3
	Teacher	5	8.2
Last 12 months sexual practice	Yes	31	50.8
	No	30	49.2
Age at first sexual practice	15-17	29	47.5
	18 -21	32	52.5
Condom use during first sex	Yes	44	72.1
	No	17	27.9

**Figure 4:**
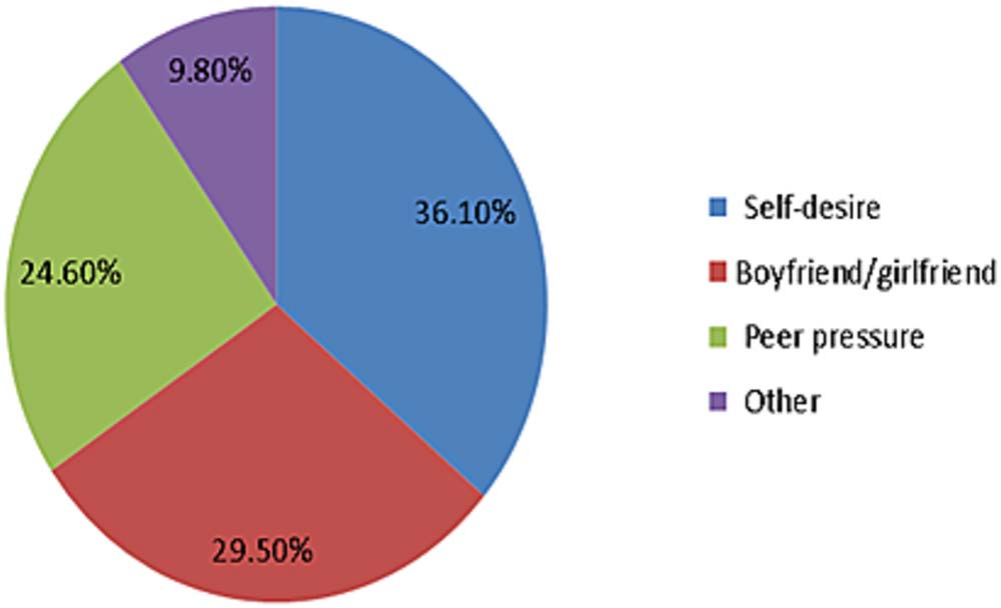
Reason for sexual activity of secondary school student in Addis Ketema sub-city, 2021.

#### Behavioral and contextual characteristics

Among all respondents, 19.5% reported alcohol consumption, 14.7% had watched pornography, 4.5% chewed khat, and 3.4% smoked cigarettes. Peer pressure was cited as an initiating factor by 68.9% of sexually active adolescents. Overall, 62.6% of students discussed sexual issues; of these, 57.5% talked with friends while only 19.5% discussed with family. An overwhelming majority (92.1%) believed reproductive health education is necessary, with 60.9% indicating it should be provided in schools. Social media usage was nearly universal (90.1%), and among users, 54.2% reported daily use (Table 4).

**Table 4:** Sexual behavior of secondary school student in Addis Ketema sub-city, 2021.

Discussion about RH issue	Yes	221	62.6%
	No	132	37.4%
Drinking alcohol	Yes	69	19.5%
	No	284	80.5%
Smoking cigarette	Yes	12	3.4%
	No	341	96.6%
Chewing khat	Yes	16	4.5%
	No	337	95.5%
Watching pornography	Yes	52	14.7%
	No	301	85.3%
Peer pressure toward sex	Yes	42	68.9%
	No	19	31.9%
Social media use	Yes	321	90.9%
	No	32	9.1%
How often do you use a social media?	Daily	174	54.2%
	Weekly	23	7.2%
	Sometimes	124	38.6%
RH education should be given?	Yes	325	92.1%
	No	28	7.9%
Preferred place to be given?	School	198	60.9%
	Home	34	10.5%
	Media	77	3.7%
	Other (youth center)	16	4.9%

### Objective 2: Factors associated with adolescent sexual activity

#### Bivariate analysis

In bivariate logistic regression, several sociodemographic and behavioral variables showed a significant association with adolescent sexual activity, including age, sex, school type, alcohol consumption, cigarette smoking, khat chewing, having a boyfriend/girlfriend, and watching pornography. These variables were subsequently entered into multivariate analysis (Table 5).

**Table 5:** Bivariate and multivariate logistic regression on factors associated with adolescent sexual activity among selected secondary schools in Addis Ketema sub-city, 2021.

**Premarital sexual practice**				**COR**	**AOR**
Sex	Male	Yes	No		
		47	124	4.548 (2.398, 8.628)	3.026 (1.257, 7.288)^a^
	Female	14	168	1	1
Age	15-17	8	97	0.303 (0.139, 0.663)	0.338 (0.111, 1.028)
	18-21	53	195	1	1
School type	Private	13	48	2.554 (1.235, 5.278)	3.153 (1.107, 8.978)^a^
	Government	28	264	1	1
Drinking alcohol	Yes	39	22	15.482 (8.124, 29.503)	5.424 (2.224, 13.228)^b^
	No	30	262	1	1
Watching pornography	Yes	38	23	32.807 (15.561, 69.167)	21.246 (7.856, 57.446)^b^
	No	14	278	1	1
Having boy or girlfriend	Yes	45	16	3.182 (1.720, 5.886)	4.577 (1.876, 12.067)^a^
	No	137	155	1	1
Smoking cigarette	Yes	7	54	7.441 (2.278, 24.309)	4.383 (0.661, 28.223)
	No	5	287	1	1
RH discussion	Yes	48	173	1	1
	No	13	119	2.540 (1.318, 4.893)	0.646 (0.246, 1.696)

COR = Crude Odds Ratio; AOR = Adjusted Odds Ratio; *p < 0.05, **p < 0.01; RH = Reproductive Health.

Abbreviation: AOR, adjusted odds ratios.

#### Multivariate analysis

After adjusting for potential confounders, the following factors remained significantly associated:

- **Sex:** Male students were approximately 3 times more likely to have engaged in sexual activity than female students (AOR = 3.03; 95% CI, 1.26-7.29).
- **Having a boyfriend/girlfriend:** Students who reported having a romantic partner were nearly 5 times more likely to be sexually active than those without a partner (AOR = 4.58; 95% CI, 1.88-12.07).
- **School type:** Students attending private schools were more than 3 times more likely to practice sexual activity compared to government school students (AOR = 3.15; 95% CI, 1.11-8.98).
- **Alcohol consumption:** Students who consumed alcohol were 5.4 times more likely to be sexually active than nonconsumers (AOR = 5.42; 95% CI, 2.22-13.23).
- **Pornography viewing:** Students who watched pornography were 21.2 times more likely to have engaged in sexual activity compared to those who did not (AOR = 21.25; 95% CI, 7.85-57.45).

## Discussion

The overall prevalence of adolescent sexual activity in this study was 17.3% (95% CI, 13.5-21.6), with 50.8% of sexually active students reporting engagement within the preceding 12 months. This finding is comparable to studies conducted in Gojam (19%), Adigrat (17.6%), and Kolfe Keraniyo sub-city (19.7%),^4^^,^^8^^,^^9^ all of which sampled school-going adolescents in predominantly urban or semi-urban settings with similar age distributions (mean age 16-18 years). In contrast, higher prevalence rates have been reported in Jimma (28.2%), Debre Markos (31.3%), Nekemte (48.6%), Goba town (31.2%), and Gondar (26.7%).^5–7^^,^^10^^,^^11^ These differences may reflect not only study period (the present data were collected in 2021, whereas many earlier studies were conducted between 2015 and 2019) but also regional socioeconomic and cultural characteristics. For instance, Nekemte and Goba are towns with more agrarian economies and traditionally conservative norms; the higher reported rates there may be partly due to different definitions of “sexual activity” or to higher inclusion of out-of-school youth. Notably, our prevalence is higher than the national Ethiopia Demographic and Health Survey (EDHS) figure for adolescents (approximately 11%), which is expected given that school-based samples typically capture a narrower age range and the EDHS includes married youth who may report lower sexual activity due to social desirability bias.^16^

The mean age at first sexual intercourse was 17.33 years (SD = 1.28), closely aligning with findings from other Ethiopian studies (range 16.9-17.5 years),^5^^,^^6^^,^^9^^,^^17^ suggesting relative consistency in the timing of sexual debut among school-attending adolescents across diverse regions.

Regarding protective behaviors, 72.1% of sexually active students reported condom use at first sex, which is substantially higher than rates observed in Gojam (46.1%) and Mizan Aman (55.7%).^4^^,^^13^ This difference may be linked to increased exposure to school-based reproductive health clubs and peer education in Addis Ababa over the past 5 years, as well as the city’s greater access to youth-friendly health services. The primary motivations for sexual initiation were personal desire (36.1%), having a boyfriend/girlfriend (29.5%), and peer pressure (24.6%), a pattern consistent with a study in Oromia,^6^ indicating that relationship dynamics and individual agency are central drivers across regions.

### Sex as a predictor

Male students were 3 times more likely to have engaged in sexual intercourse than females (AOR = 3.03, 95% CI, 1.26-7.29). This finding mirrors results from Gojam (AOR = 3.2),^4^ Debre Markos (AOR = 2.8),^7^ and Arba Minch (AOR = 3.4).^18^ In our sample, males and females were evenly represented (50.1% vs 49.9%), yet the prevalence among males was 21.3% compared to 13.3% among females, a gap wider than reported in some rural studies where gender differences are often narrower due to earlier marriage practices. This gender disparity likely reflects persistent social norms that tolerate male sexual exploration while stigmatizing female premarital activity, a dynamic that may be more pronounced in dense urban settings like Addis Ketema where social monitoring varies by gender.

### Alcohol consumption

Students who consumed alcohol were 5.4 times more likely to be sexually active (AOR = 5.42, 95% CI, 2.22-13.23). The prevalence of alcohol use in our sample was 29.7%, comparable to studies in Hosanna (26.8%)^15^ but lower than in Nekemte (44.2%)^11^ and Goba town (38.9%).^10^ The stronger association in our study (AOR = 5.4 vs 2.8 in Nekemte and 3.9 in Goba) may be explained by the urban context where alcohol outlets are ubiquitous and adolescents have easier access. Moreover, the confluence of alcohol availability and reduced parental supervision in a densely populated, highly transient area like Addis Ketema could amplify disinhibition effects. Our AOR is, however, consistent with that reported in Hosanna (AOR = 6.0),^15^ another urban setting with similar demographic characteristics.

### Pornography exposure

Watching pornography was associated with a 21-fold increased likelihood of sexual activity (AOR = 21.25, 95% CI, 7.86-57.45). This odds ratio is notably higher than those reported in earlier studies, such as Hosanna (AOR = 3.4),^15^ Gojam (AOR = 2.3),^4^ and Gondar (AOR = 4.1).^19^ Importantly, the prevalence of pornography viewing in our sample was 48.2%, which is markedly higher than the 18%-25% reported in many regional studies.^4^^,^^15^^,^^19^ This discrepancy likely reflects the study setting: Addis Ketema was a densely populated urban area with widespread smartphone ownership, unregulated internet access, and a high density of media shops, exposing adolescents to sexually explicit content at much higher rates than in rural or smaller towns. The combination of very high exposure and the urban environment’s relative anonymity may explain the exceptionally strong association observed.

### Having a boyfriend/girlfriend and school type

Youths with a romantic partner were nearly 5 times more likely to be sexually active (AOR = 4.58, 95% CI, 1.88-12.07), consistent with studies in South Gondar (AOR = 4.3) and Nekemte (AOR = 4.7).^5^^,^^11^ Private school students were 3 times more likely to be sexually active than public school students (AOR = 3.15, 95% CI, 1.11-8.98), aligning with a study in Hawassa.^20^ The prevalence of sexual activity among private school students was 23.8% vs 14.9% in public schools. This difference may be influenced by socioeconomic factors: private school students in Addis Ketema often come from families with higher disposable income, which may translate into greater autonomy, more pocket money, and increased access to venues where sexual encounters occur. In contrast, public school students in this sub-city frequently come from lower-income households with tighter parental control.

### Factors not found significant

Unlike a study in Addis Ababa,^9^ we found no significant association between receiving pocket money and sexual activity. The prevalence of pocket money receipt was similar across studies (approximately 60%), but in our setting, pocket money may be used for transportation and meals rather than for leisure activities that facilitate sexual encounters. Peer pressure and discussions about reproductive health, which were significant predictors in other studies,^10^^,^^15^^,^^17^^,^^18^ did not reach statistical significance in our analysis. This may be because awareness campaigns in Addis Ababa over the past decade have normalized reproductive health discussions to the point that they no longer differentiate sexually active from inactive youth, or because peer dynamics in this urban context may operate differently—peer pressure may be more indirect and mediated by social media rather than direct verbal persuasion.

In summary, by comparing both the prevalence of risk behaviors and AOR across studies, and by situating findings within the distinct social, economic, and geographic contexts of the regions compared, this discussion provides a more nuanced understanding of adolescent sexual activity in Addis Ketema sub-city. The findings highlight the importance of context-specific interventions that address the high rates of alcohol use, pervasive pornography exposure, and gender-based disparities in this urban setting.

### Strengths and limitations of the study

#### Strengths

This study provides timely evidence on the prevalence and drivers of adolescent sexual activity in Addis Ketema’s secondary schools. The high response rate (98%) strengthens the findings, making them a reliable resource for educational and health policymakers to guide targeted interventions and improve reproductive health outcomes for adolescents in Ethiopia.

#### Limitations

The interpretation of these findings should consider certain methodological limitations:

1. Due to the personal nature of adolescent sexual activity, self-reported data are vulnerable to underreporting or misreporting (social desirability bias).
2. Additionally, for participants under 18 years of age, written parental consent was required. This requirement may have further intensified social desirability bias, as adolescents could have been reluctant to disclose sexual activity for fear that their parents might become aware of their participation or their answers, potentially leading to underreporting.
3. The quantitative design did not capture the nuanced contextual factors influencing sexual behavior.
4. Finally, the school-based sampling frame means the results are not representative of all adolescents in the area, excluding those not enrolled in school.

## Conclusion

A significant proportion of high school students in the study area are sexually active, with a higher prevalence among males. The primary motivations for sexual initiation were self-desire and being in a romantic relationship. Key independent predictors identified were having a boyfriend or girlfriend, alcohol consumption, pornography viewing, and attending a private school.

Public health interventions should prioritize adolescent health by addressing the behavioral, social, and environmental drivers of adolescent sexual activity. We recommend collaborative efforts between schools and health institutions to reduce substance use and educate youth on the risks of early sexual activity. Furthermore, developing community-based recreational and sports facilities could positively divert youth attention, promote healthier lifestyles, and support their overall mental and social well-being.

## Data Availability

The datasets generated during and/or analyzed during the current study are available from the corresponding author on reasonable request.
